# Removal of choroidal vasculature using concurrently applied ultrasound bursts and nanosecond laser pulses

**DOI:** 10.1038/s41598-018-31045-w

**Published:** 2018-08-27

**Authors:** Haonan Zhang, Xinyi Xie, Jia Li, Yu Qin, Wei Zhang, Qian Cheng, Songtao Yuan, Qinghuai Liu, Yannis M. Paulus, Xueding Wang, Xinmai Yang

**Affiliations:** 1 Department of Biomedical Engineering, University of Michigan, Ann Arbor, MI USA; 2 Institute of Acoustics, School of Physics Science and Engineering, Tongji University, Shanghai, China; 3 Department of Ophthalmology, the First Affiliated Hospital of Nanjing Medical University, Nanjing, P.R. China; 4 Department of Ophthalmology and Visual Sciences, University of Michigan, Ann Arbor, MI USA; 5 Institute for Bioengineering Research and Department of Mechanical Engineering, University of Kansas, Lawrence, KS USA

## Abstract

Pathologic microvasculature plays a crucial role in innumerable diseases causing death and major organ impairment. A major clinical challenge is the development of selective therapies to remove these diseased microvessels without damaging surrounding tissue. This report describes our development of novel photo-mediated ultrasound therapy (PUT) technology for precisely removing choroidal blood vessels in the eye. PUT selectively removes microvessels by concurrently applying nanosecond laser pulses with ultrasound bursts. In PUT experiments on rabbit eyes *in vivo*, we applied 55–75 mJ/cm^2^ of light fluence at the retinochoroidal surface at 532-nm and 0.5 MPa of ultrasound pressure at 0.5 MHz. PUT resulted in significantly reduced blood perfusion in the choroidal layer which persisted to four weeks without causing collateral tissue damage, demonstrating that PUT is capable of removing choroidal microvasculature safely and effectively. With its unique advantages, PUT holds potential for the clinical management of eye diseases associated with microvessels and neovascularization.

## Introduction

The ability to precisely remove pathological microvessels can significantly impact a number of conditions such as cancer, inflammation, and eye diseases which are characterized by neovascularization and increased vascular permeability. Several antivascular therapies^1–4^ have been developed to treat microvessels in these conditions and improve the prognosis by controlling the progression of neovascularization. The development of these antivascular therapies has particularly benefited some of the leading causes of blindness, such as wet age-related macular degeneration (AMD) and diabetic retinopathy (DR). In these diseases, ocular neovascularization, or abnormal new blood vessels, can result in rapid and irreversible vision loss. Removing these new blood vessels has been the focus of therapeutic strategies including pharmacologic, laser, mechanical, and even surgical techniques. Current treatment involves frequent (often monthly) intravitreal injections of anti-vascular endothelial growth factor (VEGF) agents, which cause neovascularization regression and vascular permeability reduction. These monthly treatments represent a huge burden on elderly patients, their families, physicians, and the healthcare system. Furthermore, despite monthly treatment with anti-VEGF, many patients continue to have persistent disease activity (PDA). A recent study focused on the long-term efficacy of anti-VEGF therapy found that despite monthly anti-VEGF therapy, 20% of patients become legally blind and another 30% suffer from some degree of vision loss after 5 years^5^.

For patients who respond poorly to anti-VEGF therapy, alternative therapies must be sought to prevent them from becoming blind. One alternative option available is photodynamic therapy (PDT)^1^. However, PDT has serious side-effects which limit its clinical effectiveness. PDT requires the systemic injection of photosensitizer, which makes the skin and eyes sensitive to light after the treatment and requires the patient to avoid sun exposure for several days. PDT may also cause a photosensitivity reaction, dye extravasation, transient visual disturbances, infusion-related back pain, and even choroidal infarction resulting in acute, severe vision loss^6^. PDT is both time and labor-intensive, requiring patient dose-dependent calculations, slow intravenous infusion, and time-sensitive administration which can be very disruptive in a busy clinic.

Laser photocoagulation, which depends on the heat generated through light absorption by tissue, is another technique to remove microvasculature^2–4^ in the eye. Due to the use of high laser energy and millisecond pulse duration, damage can occur to the normal surrounding cells, such as the neurons (photoreceptor) which are adjacent to the retinal pigment epithelium (RPE), and 150 µm away from the retinal vasculature, resulting in serious complications such as retinal atrophy, thinning in the regions of laser application, paracentral scotomas^7^ which can enlarge over time^8^, subretinal neovascularization^9^, and subretinal fibrosis^10^. These side effects from conventional laser photocoagulation can significantly affect a patient’s quality of life.

By combining laser and ultrasound, we have recently developed a non-invasive photo-mediated ultrasound therapy (PUT) technique to shut down microvessels^11–13^. PUT is fundamentally different from any of the existing laser or ultrasound-based antivascular technologies including PDT, photothermal therapy, photothermolysis, and antivascular ultrasound therapy. PUT utilizes cavitation-based method to permanently shut down microvessels, and the cavitation produced by PUT is based on the novel photoacoustic cavitation mechanism^11^. More importantly, PUT utilizes concurrently applied ultrasound bursts and laser pulses, both are safe to biological tissues. In PUT, only optically absorbing vessels illuminated concurrently by the light pulse and the ultrasound burst are treated. Therefore, the treatment is a joint effect between the low-fluence light and the low-energy ultrasound, meaning that neither light nor ultrasound alone can produce any damage in any tissue.

During PUT, cavitation activity is promoted precisely in the targeted microvessels. The mechanical stresses produced by oscillating cavitation bubbles in the microvessels can directly impact the physiological functions of endothelial cells, red blood cells and platelets, resulting in vasocontraction, blood clot formation and hemorrhage^14–21^. The mechanism is similar to that of PDT, where microvessels are also destroyed by triggering the physiological functions of endothelial cells, red blood cells and platelets. However, unlike PDT which involves a photosensitizer, PUT is completely non-invasive and agent-free, and can offer high treatment precision. This technique has the potential to non-invasively shut down diseased microvessels to achieve optimal treatment outcome for patients^11^.

In this study, we developed a system based on PUT technology to facilitate removal of microvessels in the choroid of the eye. The capability of this system in shutting down choroidal vessels was tested on a rabbit eye model, and the safety for ophthalmological applications was examined via subsequent histopathological analyses.

## Results

**Figure 1:**
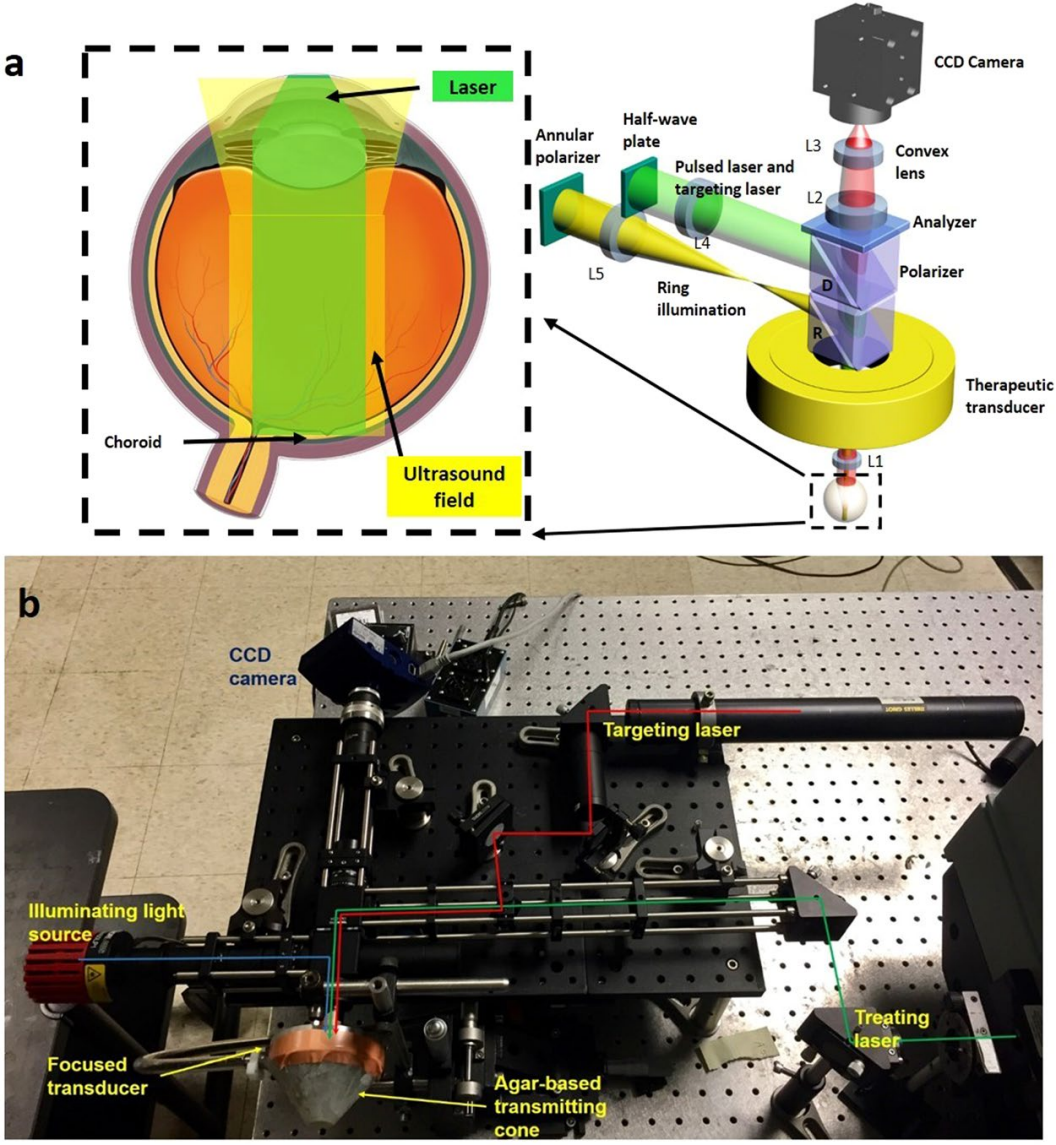
System schematic. (**a**) System schematic for PUT treatment of rabbit eye. (**b**) Photograph of the PUT treatment system. D: dichroic cube. R: beam-splitter. L1–L5: lens.

Using the experimental setup shown in Fig. 1, PUT was performed on the rabbit choroid. For all the cases in PUT treated group, the ultrasound pressure applied on the choroid was 0.5 MPa at 0.5 MHz; while the applied laser light fluence at the choroidal surface ranged from 55 to 75 mJ/cm^2^ and was titrated to achieve blood vessel blurring immediately post treatment.

**Figure 2:**
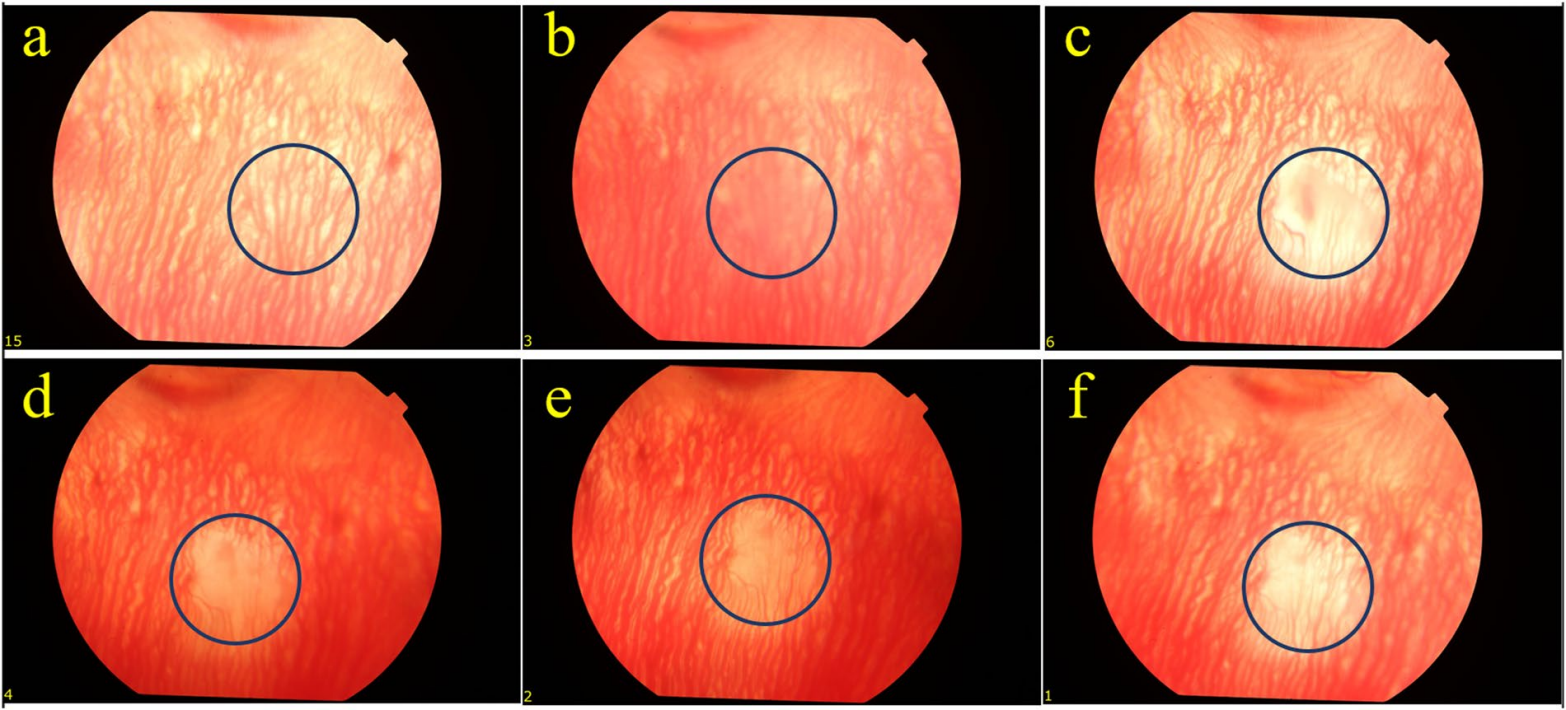
Fundus photographs following a single PUT treatment on a rabbit choroid up to 4 weeks after the treatment. Blue circles indicate the treated area. (**a**) The fundus photo taken before the PUT treatment. (**b**) The fundus photo taken immediately after the PUT treatment. The blood vessel margins in the treated area appeared blurry. (**c**) The fundus photo taken at 1 week after the PUT treatment. Pallor occurred in the region of treatment with largely diminished choroidal vessels. (**d**–**f**) The photos taken at 2, 3, and 4 weeks, respectively, after the PUT treatment. The reduction in choroidal vessels persisted until week 4. Ultrasound: 0.5 MPa at 0.5 MHz; Laser: 75 mJ/cm^2^ at retina surface.

Figure 2 shows fundus photographs of the choroid in a rabbit eye before and after PUT treatment, which were obtained through a fundus camera. The fundus photography before PUT treatment in Fig. 2a shows a rich layer of blood vessels in the treated region. Immediately (less than 1 minute) after PUT treatment, the blood vessel margins in the treated region appeared blurry on the fundus photography in Fig. 2b, likely due to an increase in vascular permeability obscuring the vessel margins. By 1 week, pallor occurred in the region of treatment with greatly diminished choroidal vessels as shown in Fig. 2c. The area with diminished choroidal blood vessels persisted to 4 weeks as demonstrated in Fig. 2d–f.

**Figure 3:**
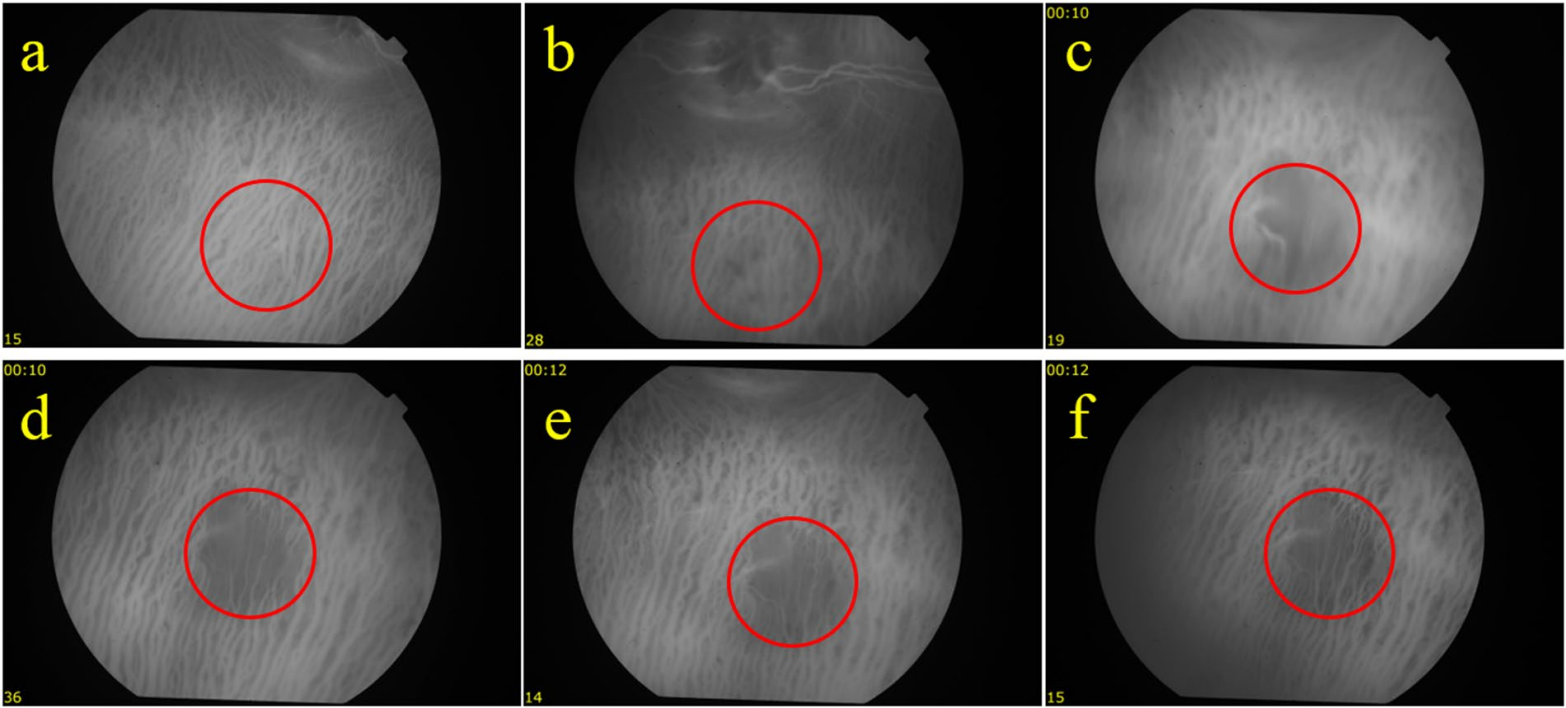
ICGA following a single PUT treatment on a rabbit choroid up to 4 weeks after treatment. (**a**) Indocyanine green angiography (ICGA) taken before the PUT treatment. (**b**) ICGA taken right after the PUT treatment, showing slightly reduced blood perfusion in the treated area. (**c**–**f**) ICGA taken at 1 week, 2 weeks, 3 weeks, and 4 weeks, respectively, after the PUT treatment, demonstrating nonperfusion in the treated area which persisted until week 4. Red circles indicate treated area.

**Figure 4:**
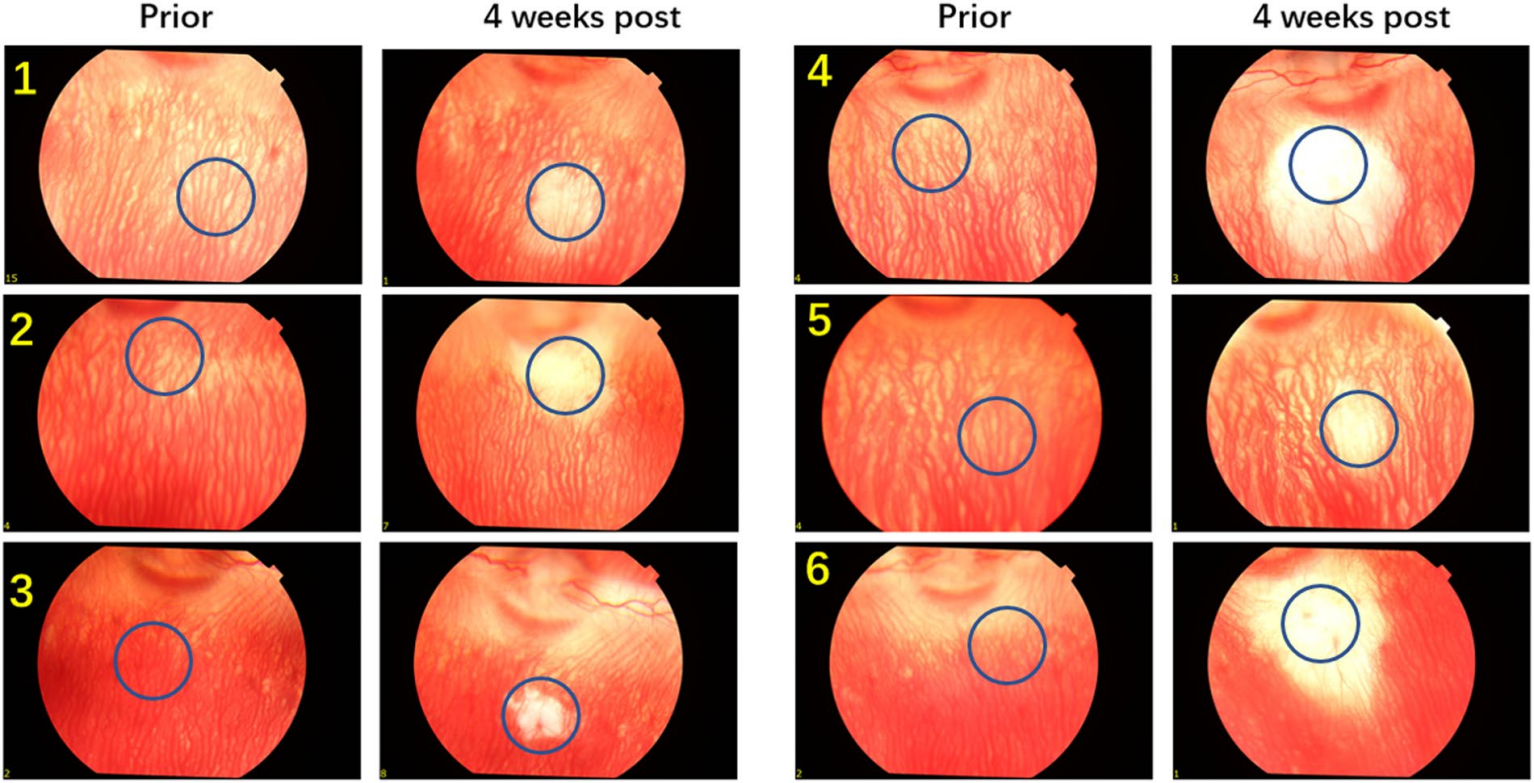
Fundus photographs of rabbit choroid before and 4-week after PUT treatment (6 cases). Blue circles indicate the treated areas. This demonstrates in 6 rabbits reproducible choroidal nonperfusion in the treated area which persisted until week 4. Ultrasound: 0.5 MPa at 0.5 MHz; Laser: 75 mJ/cm^2^ for rabbit 1,: 60 mJ/cm^2^ for rabbit 2, and 55 mJ/cm^2^ for rabbit 3–6. All parameters were estimated on the retina surface.

To further confirm the reduction in blood perfusion in the choroidal layer, indocyanine green angiography (ICGA) was performed. Figure 3 shows the ICGA before and after PUT treatment. In comparison with Fig. 3a, which is the ICGA before PUT treatment, Fig. 3b shows slightly reduced blood perfusion in the treated region at 15 minutes after PUT treatment. However, significantly reduced blood perfusion in the treated region was noticed one week after treatment. The reduction in blood perfusion persisted to 4 weeks (Fig. 3d–f). At 4 weeks after PUT, the reduced blood perfusion in the treated region was largely persisted with some of relatively large vessels started to show signs of slight re-perfusion, as shown in both Figs 2f and 3f. The experiment was repeated on six rabbits with the results shown in Fig. 4. In each case, the size of the treated area was controlled by the laser beam on the choroidal layer. By comparing the fundus photographs acquired before and 4 weeks after the treatment, we can see that PUT efficiently removed the choroidal blood vessels with various sizes.

**Figure 5:**
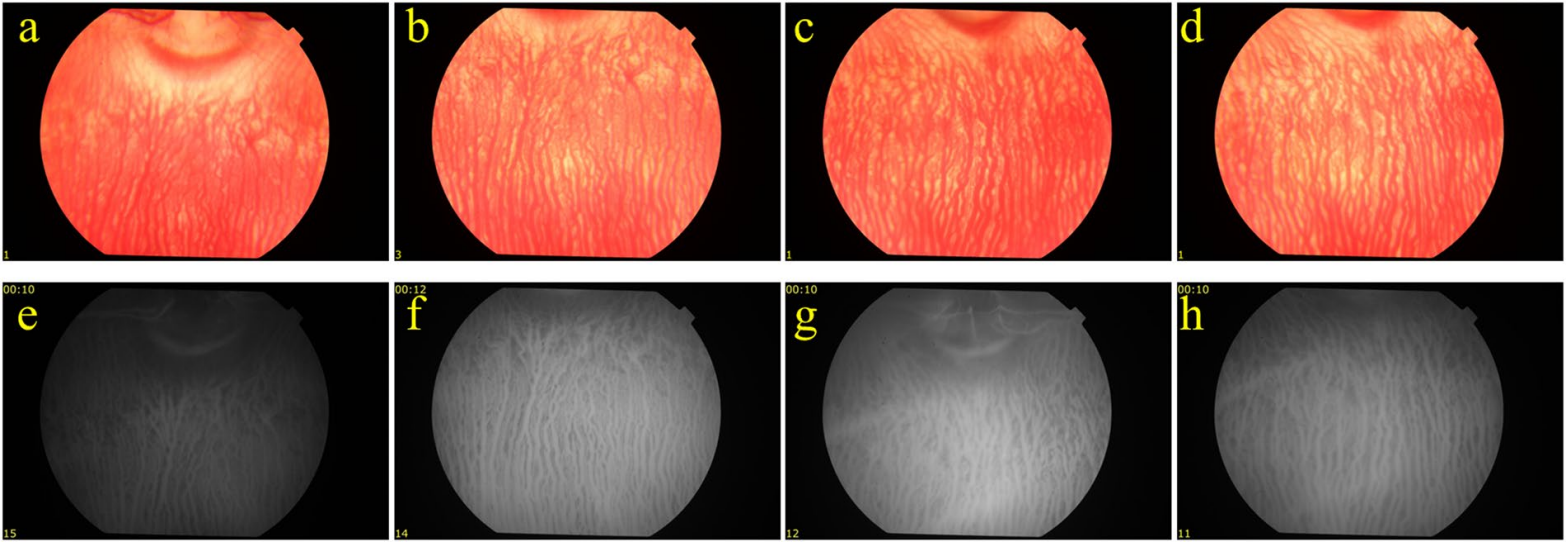
Fundus photographs and ICGA for the control cases where either only laser or only ultrasound was applied to the choroid layer. (**a**,**b**) Color fundus photos taken from a rabbit eye before and 4 weeks after the treatment using 75 mJ/cm^2^ laser pulses (no ultrasound burst applied). (**c**) and (**d**) Color fundus photos taken from a rabbit eye before and 4 weeks after the treatment using 0.5 MPa ultrasound bursts (no laser pulse applied). (**e**,**f**) ICGA results correspondent to the photos in (**a**,**b**) respectively. (**g**,**h**) ICGA results correspondent to the photos in (**c**,**d**) respectively.

Figure 5 shows the examples of fundus photography and ICGA results for the control groups, where either laser-only or ultrasound-only treatment was employed. For laser-only group, the applied laser light fluence at the choroidal surface was 75 mJ/cm^2^. For ultrasound-only group, the ultrasound pressure applied on the choroid was 0.5 MPa at 0.5 MHz. For all the cases, the blood vessel size and density remained stable during the 4-week observation period, and no choroidal vessel reduction was noticed in the treatment area, demonstrating that vessel reduction after the PUT treatment was due to the synergistic effect of laser pulses and ultrasound bursts.

**Figure 6:**
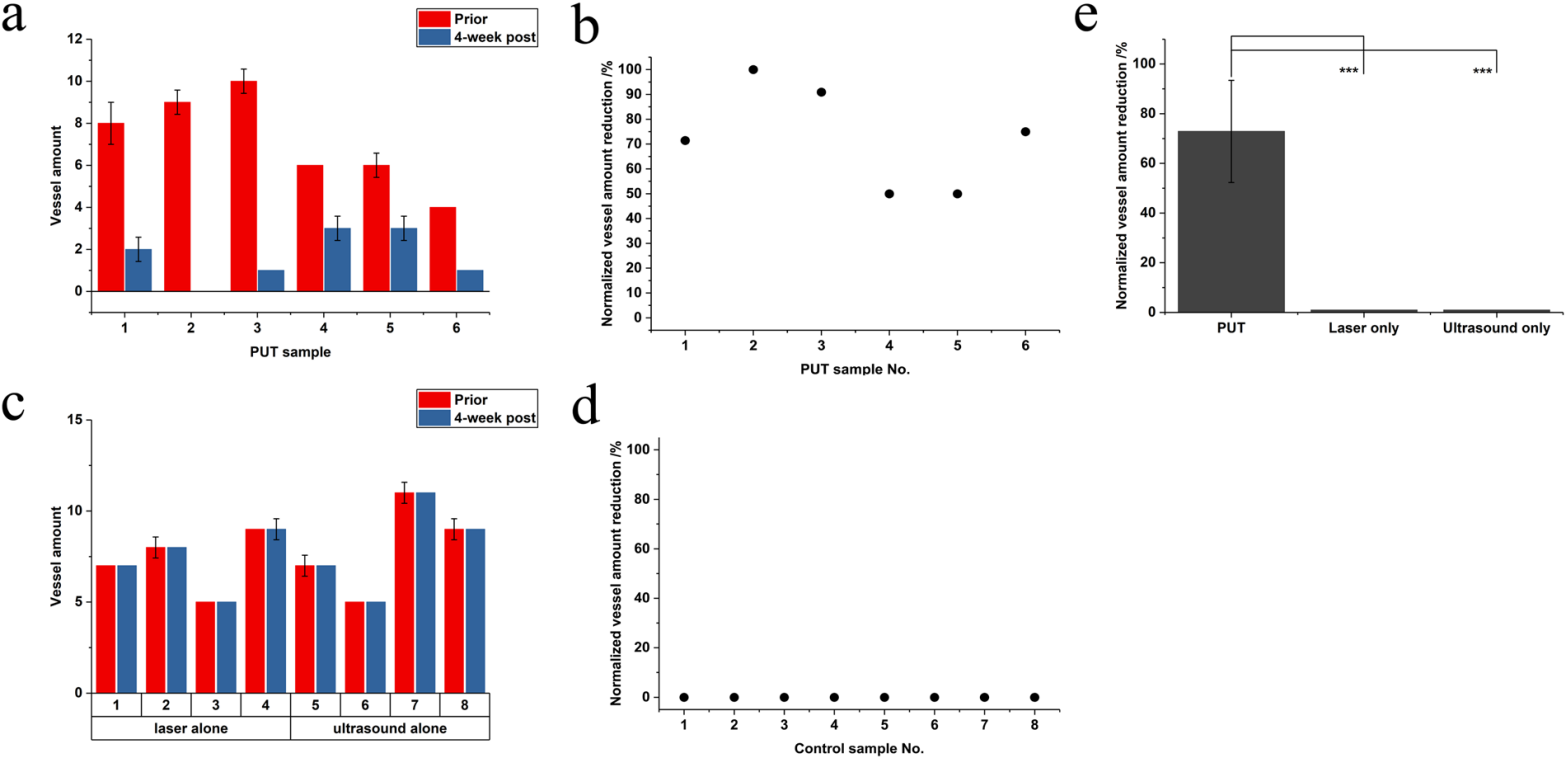
Statistical analysis of the reduction in vessel amount in choroidal layer in response to treatment. (**a**) The numbers of counted vessels inside the treated area before and 4 weeks after the PUT treatment for all the six cases. Error bars (median ± standard error) were calculated based on three fundus pictures for each eye at the same time point. (**b**) Normalized vessel amount reduction of the PUT group. (**c**) The numbers of counted vessels inside the treated area before and 4 weeks after the treatment using either laser only or ultrasound only (i.e., control groups). Error bars (median ± standard error) were calculated based on three fundus pictures for each eye at the same time point. (**d**) Normalized vessel amount reduction of the control group. (**e**) Percentile reduction in vessel amount in response to different treatments (i.e., PUT, laser only, and ultrasound only). *** stands for p < 0.01.

To quantitatively evaluate the treatment efficacy, the number of choroidal blood vessels in the treated region in each eye was counted to quantify the local perfusion change in response to PUT. Figure 6a shows the numbers of counted blood vessels inside the treated areas before and 4-week after the PUT treatment for all the six rabbits. Figure 6b shows the relative changes in blood vessel numbers, which was computed by (100 × $\frac{prior-4\,week\_post}{prior}$)%. In the six cases treated by PUT, the numbers of blood vessels in the treated areas were all reduced significantly. Figure 6c shows the quantitative results for the control groups, including 4 cases treated by ultrasound-only without applying laser pulses and 4 cases treated by the laser-only without applying ultrasound bursts. The relative changes in blood vessel numbers are shown in Fig. 6d, which are essentially all 0 because no change was observed. Figure 6e compares the average relative changes between PUT group and control groups. On average, a 72.89 ± 20.56% reduction in the number of blood vessels were achieved in PUT group, which was statistically significant compared with the control groups (p < 0.01, for a paired t-test). At the same time, the differences between PUT group and either of the rest of 2 groups (laser only and ultrasound only) were statistically significant (p < 0.01, for a Fisher’s exact test).

**Figure 7:**
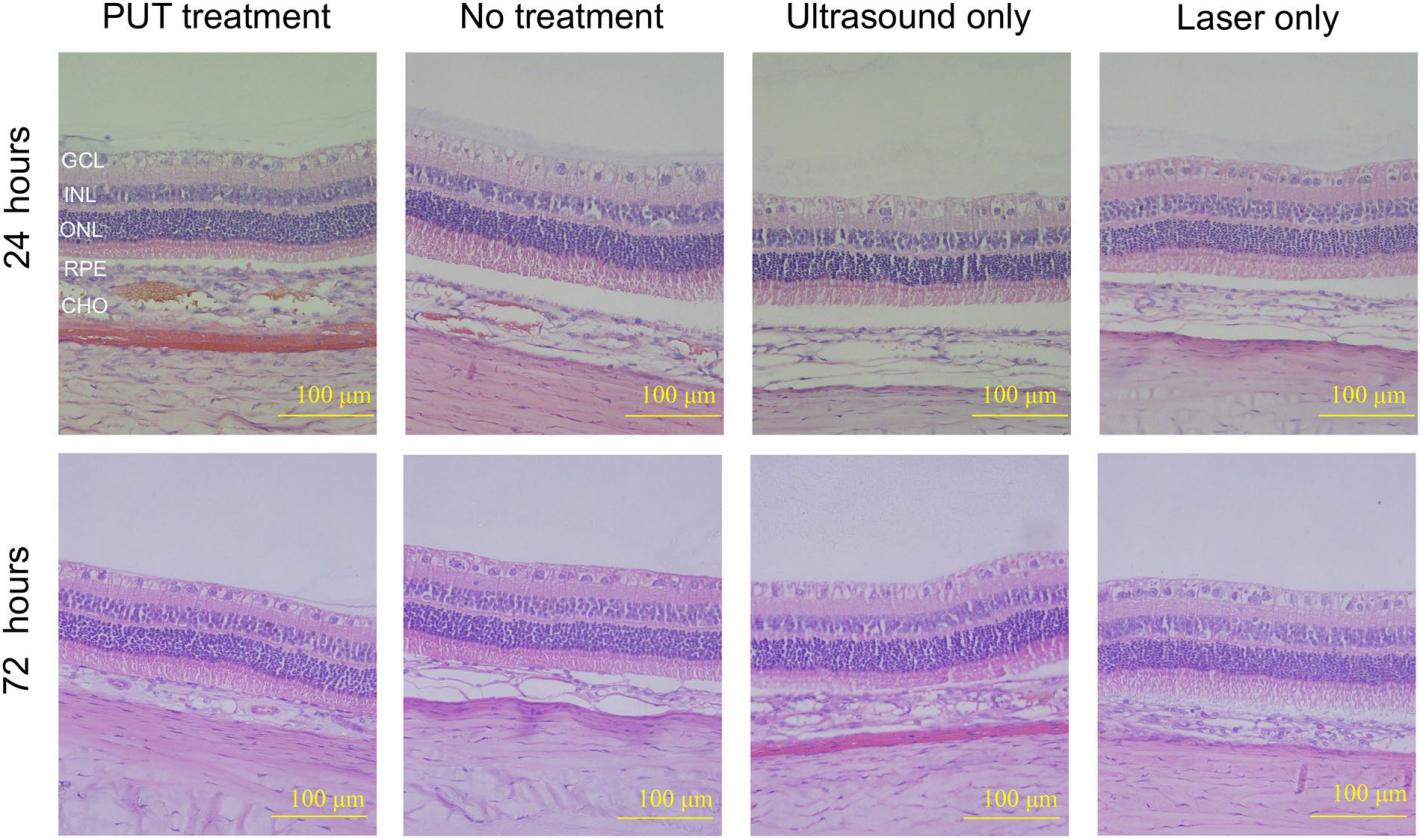
Histological results to demonstrate the safety of PUT. Representative H& E stained histological sections show the bottom layers of the rabbit eyes in different groups at different time points (24 hours and 72 hours) after the treatment (40× amplification). For all the cases including those in the PUT group and those in the control groups (no treatment, laser only, and ultrasound only), no morphological damage was observed at either 24 or 72 hours after treatment. In the histological section at 24 hours after PUT treatment, possible blood clots were noticed in the treated choroidal blood vessels. Ultrasound: 0.5 MPa at 0.5 MHz; Laser: 75 mJ/cm^2^ at retina surface. GCL: ganglion cell layer, INL: inner nuclear layer, ONL: outer nuclear layer, RPE: retinal pigment epithelium, CHO: choroid.

Standard H&E histological results from the rabbit eyes, as shown in Fig. 7, confirmed the safety of PUT. In the histological slides obtained at 24 hours and 72 hours after the PUT treatment, the retinal and the choroidal tissue structures were integrated, continuous, and normal. No morphological damage in any tissue layer can be noticed. In addition, in the section at 24 hours after PUT treatment, possible blood clots were noticed in the treated choroidal blood vessels. Blood clots in the choroidal vessels could not be found in any of the control cases which were treated either by laser pulses only (with the same light fluence) without applying ultrasound bursts or by ultrasound bursts only (with the same ultrasound pressure) without applying laser pulses.

**Figure 8:**
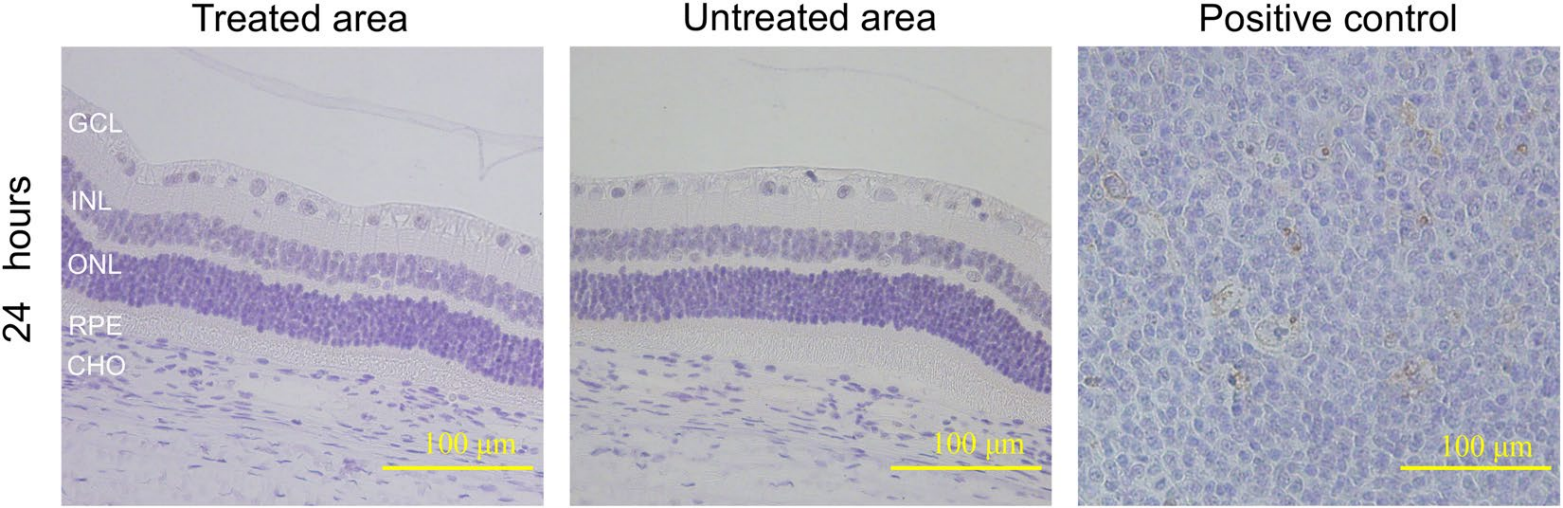
TUNEL assay showing no apoptosis after PUT. Representative photos of TUNEL assay of treated area and untreated area in the rabbit eye taken at 24 hours after PUT treatment are shown (40× amplification). Section of human tonsil tissue was used as positive control for TUNEL staining. Brown staining cells indicate TUNEL-positive cells. Neither the treated area nor the untreated area showed TUNEL-positive cells in the retina and the choroid. Ultrasound: 0.5 MPa at 0.5 MHz; Laser: 75 mJ/cm^2^ at retina surface. GCL: ganglion cell layer, INL: inner nuclear layer, ONL: outer nuclear layer, RPE: retinal pigment epithelium, CHO: choroid.

The finding from the TUNEL assay, as shown in Fig. 8, further confirmed the safety of PUT, and demonstrated that the retinal and choroidal tissues were left intact. The TUNEL assay carried out at 24 hours after PUT treatment (time for cell apoptotic peak in conventional laser therapy) showed no TUNEL-positive cells in the treated area, suggesting that PUT of choroid vessels did not lead to cell apoptosis in surrounding tissues^22^.

## Discussion

We have demonstrated that PUT, as a newly developed antivascular therapeutic method^11^, can non-invasively eliminate microvessels in the choroid in rabbit eyes with excellent efficacy and safety. In comparison with the standard anti-VEGF therapy for wet AMD, PUT removes microvessels through a different mechanism, which is to directly damage vascular endothelial cells through mechanical forces produced via cavitation^11^. Through a single treatment, PUT can largely stop the perfusion in the target area. The shut down in perfusion can last up to 4 weeks without obvious revascularization. Suspicious vascular changes were observed on some of the eyes after 4 weeks, indicating that revascularization might occur in a longer period. We noted that the standard anti-VEGF therapy for wet AMD needs to be performed monthly because of revascularization. PUT may be applied in a similar fashion to remove choroidal microvessels if revascularization occurs. On the other hand, we expect that the potential revascularization will be related to the applied laser and ultrasound parameters. Further study on the optimization of the applied laser and ultrasound parameters to minimize revascularization will be needed in the future. In comparison with traditional laser photocoagulation, PUT can treat microvessels without damaging surrounding tissues. By taking advantage of the high intrinsic optical contrast between hemoglobin and other tissues, the treatment effect of PUT is limited to blood vessels only, and unwanted damage to surrounding tissues is minimized.

In comparison with PDT, PUT is an agent-free technique. The agent-free feature has huge clinical benefits, as it not only improves the safety but also reduces time and labor involved in treatment, making the procedure fit better in patient workflow. Our study on rabbit eyes *in vivo* demonstrated that PUT working with 0.5 MPa at 0.5 MHz for ultrasound bursts and 55 mJ/cm^2^ for light pulses could remove microvessels efficiently. The 0.5 MPa ultrasound is much lower than the cavitation threshold (~4 MPa) reported in the literature^23^; while the 55 mJ/cm^2^ light fluence is much lower than that needed for laser photocoagulation (100 to 2000 mJ/cm^2^)^24^. Therefore, PUT is a synergistic effect between the nanosecond laser pulses and the ultrasound bursts. This synergy between light and sound is not only the reason for excellent selectivity but also the guarantee for the safety of PUT. In addition, because of this synergy, the outcome from PUT may be controlled precisely by adjusting the optical and the ultrasound parameters. The precision in controlling the treatment outcome can be further improved in the future by integrating PUT with real-time imaging technologies (e.g., OCT) so that image-guided treatment ensuring personalized treatment with optimal outcome can be achieved. Further studies are being pursued at evaluating the utility of PUT in selectively treating pathologic vasculature and neovascularization.

In summary, with the aforementioned unique advantages, including good efficacy and good safety in removing microvessels in the eye, PUT holds potential to be developed into a novel powerful tool for treating patients with AMD, diabetic retinopathy, and other retinal diseases. This research on PUT, as an innovative platform technique, may also open up new horizons in selective antivascular therapy across numerous disciplines. Since abnormal vasculature and neovascularization is the hallmark of many pathologic conditions, PUT may have numerous other applications such as port wine stain and cancer.

## Methods

### PUT System

The design layout of the integrated of laser and ultrasound components in the PUT system is shown in Fig. 1. A more complete schematic of the whole PUT system is presented in Supplementary Fig. S1. A standard Nd:YAG laser (Continuum Powerlite DLS 8010, Santa Clara, CA) was employed to produce laser pulses with 5-ns pulse duration and 10-Hz pulse repetition rate. The laser beam was delivered to the sample with its energy before the rabbit sclera measured by a Nova PE25BB-SH-V2 pyroelectric head (Ophir Optronics Ltd., Jerusalem, Israel). The applied laser intensity was carefully adjusted by controlling the Q-switch delay time. A 0.5-MHz therapeutic ultrasound transducer (H107, Sonic Concepts, Bothell, WA) was used to simultaneously supply ultrasound bursts which were synchronized with the laser pulses. The therapeutic transducer had a geometric focal distance of 63.2 mm, a focal depth of 21.42 mm, and a focal width of 3.02 mm. A custom-built, 3D printed cone was designed and attached to the ultrasound transducer, and filled with agar-gelatin based couplant to provide acoustic coupling. A middle hole was reserved inside the cone for the optical lens fixation and light propagation including the treatment laser beam and the illumination light beam. Both the ultrasound beam and the light beams were aligned in advance.

When the rabbit choroid was placed in the ultrasound focal zone, the ring-shaped ultrasound transducer was positioned above the eye such that the ultrasound focal plane coincided with the choroid. The central opening of the transducer allowed for the propagation of the light beams. The treatment laser light was tuned to 532 nm, one of the absorption peaks of hemoglobin. The laser beam was slightly focused, via objective lens L4 and L1, onto the front focal plane of the rabbit eye. This design ensured that the laser beam was collimated again by the eye’s optics to a beam diameter of about 1 mm. Care was taken to ensure that the ultrasound focal point (lateral spot size of about 3 mm) was concentric with the laser beam.

In order to identify the target area of interest on the choroid and evaluate morphological vascular changes during treatment in real time, a fundus imaging system was incorporated into the experimental apparatus. Imaging of the choroid was achieved using an ophthalmic lens (L1) which created an inverted image of the choroid with a magnification near unity in its back focal plane. The choroid image was then relay-imaged onto a charge coupled device (CCD) detector using lenses L2 and L3. The imaging path and the therapeutic laser path were separated using a dichroic mirror (D) that reflected a narrow band around the laser wavelength and transmitted the rest of the visible spectrum. High contrast imaging of the choroidal blood vessels was achieved by illuminating the choroid using a polarized annular-structure pattern, and focusing onto the pupil plane to minimize back-reflections from the clear ocular media. The annular illumination was relayed from an annular-shaped source via lenses L5 and the objective lens L1 and combined with the main optical path using a polarizing beam-splitter (R). R reflected the s-polarization component of the illumination light toward the rabbit eye and transmitted the p-polarized laser. An analyzer mounted on the imaging arm of the set-up was used to further improve the image contrast by cleaning up any remainder s-polarized light specularly reflected from the various interfaces. A targeting laser beam that overlaid the treatment laser beam was also added. The targeting laser beam allowed the identification of the exact treatment location on the choroid through the CCD before PUT.

The laser system supplied laser pulses with light fluence at the choroid precisely controlled. Each laser pulse was delivered to the target area to overlay the beginning phase of each ultrasound burst^11^. A function generator (HP33250A, Agilent Technologies, Santa Clara, CA) was triggered by the laser system to produce 0.5 MHz bursts (10-ms long) with 10% duty cycle at a pulse repetition rate of 10 Hz. The signals from the function generator were then amplified by a 50-dB radio frequency amplifier (240 L, ENI Technology, Inc., Rochester, NY) before being sent to the therapeutic ultrasound transducer. The peak negative ultrasound pressure at the position of choroid was measured *in situ* in an euthanized rabbit eye by using a calibrated needle hydrophone (HNC-1500, Onda, Sunnyvale, CA). During treatment, cavitation occurred when the laser pulse and the beginning of the ultrasound burst were synergistically overlaid. The remaining portion of the ultrasound burst further drove the formed bubbles to stimulate the endothelial cells in the blood vessels.

### *In vivo* Rabbit Model

New Zealand white rabbits (2.5 to 3.0 kg, 3 to 4 months old, both genders) were acquired from the Center for Advanced Models for Translational Sciences and Therapeutics at the University of Michigan Medical School. The animals were housed in an air-conditioned room with a 12-hour light–dark cycle, fed standard laboratory food, and allowed free access to water. All the animal handling procedures were carried out in compliance with protocols approved by the Institutional Animal Care and Use Committee (IACUC) at the University of Michigan (Protocol number PRO00006487, PI Paulus), with strict adherence to the ARVO Statement for the Use of Animals in Ophthalmic and Vision Research.

Twenty rabbits were randomly assigned to three groups as follows: group 1, PUT treatment (n = 8, two animals were euthanized after treatment for histological analysis); group 2, laser only (n = 6, two animals were euthanized after treatment for histological analysis); group 3, ultrasound only (n = 6, two animals were euthanized after treatment for histological analysis). The sample sizes were confirmed by a statistical power analysis (with the null hypothesis that 0% of vessels would be reduced by PUT, estimating 50% as average PUT reduction and 20% as standard deviation of PUT reduction, the statistical power of current model is beyond 99%). Only the left eye of each animal was used. Each rabbit was anesthetized with a combination of ketamine hydrochloride 40 mg/kg (Ketalar®; Par Pharmaceutical Co Inc, Spring Valley, NY) and xylazine 5 mg/kg (Anased®; MWI/VetOne, Boise, ID) injected intramuscularly (IM) to induce anesthesia and analgesia. The pupils were dilated with 1 drop each of tropicamide 1% ophthalmic and phenylephrine 2.5% ophthalmic. Tetracaine hydrochloride 1% was administered as a topical anesthetic to both eyes of each animal prior to experiments. The choroidal layer (approximately one disc diameter from the edge of the optic disc) was then exposed to multiple powers of laser plus ultrasound, laser only control, and ultrasound only control. Color fundus images and indocyanine green angiography (ICGA) images were acquired before, immediately after, and weekly following PUT treatment using digital fundus camera (Topcon 50EX Fundus Camera and Digital Imaging System, Topcon, Tokyo, Japan). ICGA was performed by injecting 0.2 ml/kg of ICG (HUB pharmaceuticals LLC, NDC 17238-424-06, Rancho Cucamonga, CA) intravenously (IV) into the marginal ear vein. At the end of each experiment, the rabbit was euthanized with a lethal dose of Beuthanasia-D (Merck Animal Health; Summit, NJ, USA) IV through ear vein injection along with the removal of a vital organ.

### Image Processing

Image processing was performed to quantify the change in vessel amount in the treated area in choroidal layer post PUT treatment. To correctly count the number of vessels, a Wellner’s self-adaptive thresholding algorithm^25^ was used to enhance the contrast between the blood vessels and the surrounding tissues in each color fundus image. Then the number of vessels inside the treated area was counted for both the fundus image acquired before the treatment and the fundus image acquired at 4-week after the treatment.

### Histology

The rabbits from the PUT treated group and the control groups, along with 2 rabbits without any treatment were euthanized at 24 hours and 72 hours after treatment. The eye was immediately enucleated carefully and fixed in Davidson’s Solution for 24 hours, followed by transferred to 50% ethanol for 8 hours and then preserved in 70% ethanol until processing. After removal of the anterior segment and the vitreous fluid, the eyecup containing the treated area was dissected into multiple representative strips with a thickness of 5 mm. All the strips were pre-fixed in 4% agar and then embedded in paraffin. The paraffin-embedded sections (thickness 5 μm) were obtained with a microtome and stained with hematoxylin and eosin (H&E). Theses sections were analyzed under a light microscope (Olympus BX-51). Photographs were taken with a digital camera (Olympus DP70).

### TUNEL Assay

To further confirm the safety, terminal deoxynucleotidyl transferase (TdT) dUTP nick-end labeling (TUNEL) assay was performed to detect cell apoptosis. TUNEL staining assay was performed using ApopTag Peroxidase *In Situ* Apoptosis Detection kit (Chemicon International, Inc., San Francisco, CA) and was carried out according to the manufacturer’s instruction. Briefly, deparaffinized chorioretinal sections were prepared as described and digested using proteinase K. After quenching of endogenous peroxidase with hydrogen peroxide using 3.0% hydrogen peroxide in PBS, sections were then placed in an equilibration buffer and incubated with digoxigenin-conjugated dUTP in a terminal deoxynucleotidyl transferase-catalyzed reaction. The reaction was terminated with stop/wash buffer, provided with the kit. Sections were then stained with a 3, 3′-diaminobenzidine (DAB) followed by counterstaining with hematoxylin. The sections were evaluated under a light microscope (Olympus BX-51).

### Statistical analyses

Statistical analyses were performed using SPSS software version 24.0 (SPSS Inc., Chicago, IL). Paired t-test was used to compare the reduction of vessel amount between the PUT group and the control groups. The Fisher’s exact test or χ^2^ test was used for comparisons of categorical data between groups. Data are expressed as mean ± standard error of the mean. A 2-tailed P-value of <0.05 was used to indicate the statistical significance.

## Electronic supplementary material

Supplementary Information

## Data Availability

The datasets generated and analyzed during the current study are available from the corresponding authors on reasonable request.
