# Supplementary material for: Removal of choroidal vasculature using concurrently applied ultrasound bursts and nanosecond laser pulses
Source: Sci Rep. 2018 Aug 27;8:12848. doi: 10.1038/s41598-018-31045-w (PMC6110758; doi:10.1038/s41598-018-31045-w)
Supplement: Supplementary file 1 — Supplementary Information [file 41598_2018_31045_MOESM1_ESM.docx]

**Supplementary Information**

**Removal of choroidal vasculature using concurrently applied ultrasound bursts and nanosecond laser pulses**

Haonan Zhang,^1,2, §^ Xinyi Xie,^1,3, §^ Jia Li,^1,3, §^ Yu Qin,^1,2^ Wei Zhang,^1^ Qian Cheng,^2^ Songtao Yuan,^3^ Qinghuai Liu,^3^ Yannis M. Paulus,^1,4,*^ Xueding Wang,^1,2,*^ Xinmai Yang^5,*^

^1^ Department of Biomedical Engineering, University of Michigan, Ann Arbor, MI, USA

^2^ Institute of Acoustics, School of Physics Science and Engineering, Tongji University, Shanghai, China

^3^ Department of Ophthalmology, the First Affiliated Hospital of Nanjing Medical University, Nanjing, P.R. China

^4^ Department of Ophthalmology and Visual Sciences, University of Michigan, Ann Arbor, MI, USA

^5^ Institute for Bioengineering Research and Department of Mechanical Engineering, University of Kansas, Lawrence, KS, USA

^§^These authors contributed equally.


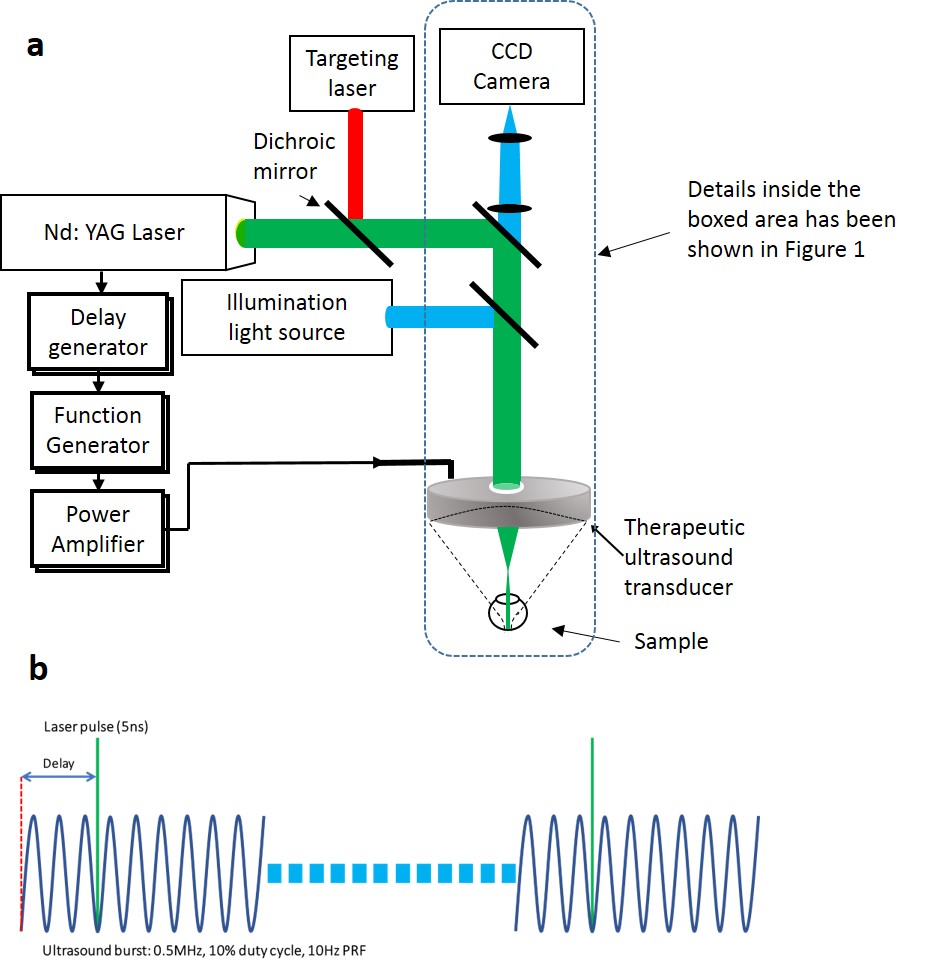


**Supplementary Figure S1** | (a) Schematic of the overall PUT treatment system for the rabbit eye; (b) time sequence of laser pulses and ultrasound bursts.
